# Colony-PCR Is a Rapid Method for DNA Amplification of Hyphomycetes

**DOI:** 10.3390/jof2020012

**Published:** 2016-04-19

**Authors:** Georg Walch, Maria Knapp, Georg Rainer, Ursula Peintner

**Affiliations:** Institute of Microbiology, University of Innsbruck, Innsbruck 6020, Austria; Georg.Walch@uibk.ac.at (G.W.); mca.knapp@gmail.com (M.K.); Georg.Rainer@student.uibk.ac.at (G.R.)

**Keywords:** soil fungi, direct PCR, barcoding, fungal isolates, yeasts, reference library construction

## Abstract

Fungal pure cultures identified with both classical morphological methods and through barcoding sequences are a basic requirement for reliable reference sequences in public databases. Improved techniques for an accelerated DNA barcode reference library construction will result in considerably improved sequence databases covering a wider taxonomic range. Fast, cheap, and reliable methods for obtaining DNA sequences from fungal isolates are, therefore, a valuable tool for the scientific community. Direct colony PCR was already successfully established for yeasts, but has not been evaluated for a wide range of anamorphic soil fungi up to now, and a direct amplification protocol for hyphomycetes without tissue pre-treatment has not been published so far. Here, we present a colony PCR technique directly from fungal hyphae without previous DNA extraction or other prior manipulation. Seven hundred eighty-eight fungal strains from 48 genera were tested with a success rate of 86%. PCR success varied considerably: DNA of fungi belonging to the genera *Cladosporium*, *Geomyces*, *Fusarium*, and *Mortierella* could be amplified with high success. DNA of soil-borne yeasts was always successfully amplified. *Absidia*, *Mucor*, *Trichoderma*, and *Penicillium* isolates had noticeably lower PCR success.

## 1. Introduction

Fungal pure cultures, identified with both classical morphological methods and through barcoding sequences are especially valuable for a reliable identification of environmental sequences and for comparative analyses, e.g., concerning the distribution and ecology of fungal taxa [1,2,3,4]. This, in turn, makes a fast, cheap, and reliable method for obtaining DNA sequences from fungal isolates a valuable tool.

Direct colony PCR is a fast technique, and is regularly applied for PCR amplification of bacterial cell cultures, cell lines, and yeast cultures. Moreover, direct colony PCR was also successfully established for other groups of organisms, e.g., Acanthamoeba [5,6], Chironomidae animals [7], fungus-like organisms, such as Oomycota [8], viruses [9], and plants [10]. Commercial direct PCR kits, e.g., for human tissue and blood, animals and plants, are already on the market. Yeasts and some other selected fungal taxa were successfully amplified with commercial direct PCR plant kits [10,11], but anamorphic soil fungi were not tested extensively for direct PCR success. As red yeasts have been shown to be problematic for direct PCR amplification, the method was optimized for them [12] and for selected human pathogenic yeasts, as well as for *Aspergillus fumigatus* [13]. Mutualistic Basidiomycota and Ascomycota were also successfully amplified directly from cleaned mycorrhized root tips without previous DNA extraction [14], and a direct PCR in combination with species-specific primers allowed for a fast identification of *Tuber melanosporum* fruiting bodies [15]. Fungal endophytes isolated from grapevines were successfully amplified directly from fungal colonies, but only after an intricate pre-treatment of the fungal tissue [16].

The main aim of the present study was to establish and test a modified direct colony PCR protocol for amplification of fungal tissue without laborious pre-treatment. Our second question was whether this direct colony PCR technique could be successfully applied to a wide range of important soil fungi. We, therefore, tested a wide taxonomic range of soil hyphomycetes and yeasts (123 species), and also tested for PCR reproducibility within species by including several isolates of one species in our tests.

## 2. Materials and Methods

A total of 788 fungal pure cultures from the culture collection of the University Innsbruck were used for this study. Fungal cultures were isolated from soil [17,18,19] or from wood [20]. Pure cultures of 123 soil fungal taxa were deposited in the Jena Microbial Resource Collection (JMRC). A list of tested pure cultures with morphology-based identification, collection numbers, Genbank Accession numbers, and JMRC numbers are provided in Appendix Table A1. Direct colony PCR works independently of the cultivation media and of the amplified target region [10,12,15,16], but in order to allow for a meaningful comparison of PCR success, all fungal isolates were cultivated on 3% malt extract agar (MEA) and amplified with the primers ITS1F and ITS4.

### 2.1. Media and Cultivation

PCR amplification was carried out with fungal pure cultures cultivated on 3% MEA media without antibiotics. Pure cultures were usually incubated at 25 °C, with the exception of psychrophilic fungi, which were incubated at 10 °C.

### 2.2. Morphological Identification of Isolates

Morphological identification was based on growth characteristics of cultures and on morphological characters. Additional growth media, e.g., Czapek Yeast Extract Agar (CYA) and 25% Glycerol Nitrate Agar (G25N) for *Penicillium* [21], were used to assist with morphological identification when appropriate. The use of antibiotics in growth media was omitted to avoid changes in fungal morphology that might hamper morphological identification. The identification of fungal genera was based on general literature for soil fungi [22,23]. Whenever possible, exact species identification was carried out based on monographs on the respective genera [21,24].

### 2.3. Direct PCR of Fungal Cultures

Fungal tissue for amplification was taken directly from pure cultures that were about one week old. Heat-sterilized toothpicks or sterile syringe needles were used for transferring a pin point of fungal tissue directly into the already prepared and portioned PCR reaction mixture. Care was taken to transfer only minute amounts of fungal material.

The amplification of fungal rDNA-ITS-region was carried out using the primer pair ITS1F [25] and ITS4 [26]. PCR was conducted by a Primus 96 thermal cycler (VWR Life Science Competence Center, Erlangen, Germany) in a 25 µL volume reaction containing one-fold buffer S (1.5 mM MgCl_2_, 10 mM TrisHCl, 50 mM KCl), 2 mg/mL BSA, 400 nM of each primer, 200 nM for each dNTP, and 0.75 U of Taq DNA polymerase (VWR Life Science Competence Center, Erlangen, Germany). The amplification conditions were 10 min of initial denaturation at 95 °C, followed by 30 cycles of 94 °C for 1 min, 50 °C for 30 s, and 72 °C for 1 min, and a final extension step of 72 °C for 7 min. (modified from [14]). 2 µL of PCR product from each reaction were mixed with 2 µL loading dye (six-fold diluted) and electrophoresed in a 1% (*w*/*v*) agarose gel with 10 μg/μL ethidium bromide. A GeneGenius Imaging system (Syngene, Cambridge, UK) with ultraviolet light was used for visualization. Clean-up and sequencing of PCR products was performed by MicroSynth AG (Balgach, Switzerland) with the primers ITS1 or ITS4.

### 2.4. Sequence Analysis and Data Handling

The generated rDNA ITS sequences were visualized in Sequencher (V.5.2.3; Gene Codes Corp., Ann Arbor, MI, USA) followed by BLAST analyses in GenBank and UNITE. Sequences were assembled in Sequencher to form CONTIGS with a sequence homology of 99% and an overlap of 80%. Fungal cultures with ≥99% sequence identity were defined as one molecular operational taxonomic unit (MOTU). MOTUs were used because ITS regions are sometimes not reliable for morphological species delimitation. One representative sequence of each MOTU was submitted to GenBank. Sequences can be retrieved under the GenBank accession numbers KP714530–KP714713 (also listed in Appendix Table A1).

## 3. Results

### PCR Success from Fungal Pure Cultures

**Figure 1:**
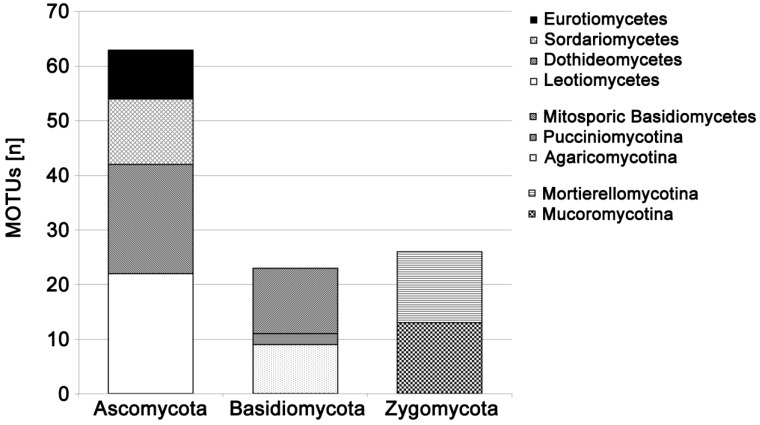
Number of fungal MOTUs tested and successfully amplified with the colony PCR technique, sorted by taxonomic affiliation.

**Figure 2:**
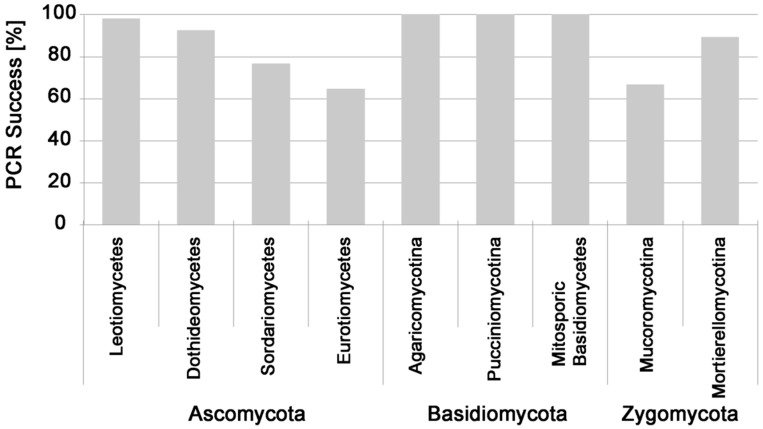
Direct PCR success for pure cultures of soil fungi belonging to different fungal subphyla.

Soil fungi belonging to Ascomycota, Basidiomycota, Mortierellomycotina, and Mucoromycotina were successfully tested (Figure 1). Direct PCR success was generally high: a total of 788 different fungal pure cultures were tested with an overall PCR success of 86%. Suitability for this direct PCR method varied between fungal groups: success was nearly 100% for soil-associated cultivable Basidiomycota, but only 67% for Mucoromycotina and 65% for Eurotiomycetes (Figure 2). This was mainly because direct PCR success of fungal cultures was characteristic for soil fungal genera: 91% of the 48 isolated genera of soil fungi had a very high (>90%, *n* = 41 genera) or high (>80%, *n* = 3 genera) PCR success, with exceptions of *Absidia* (0%), *Mucor* (58%), *Penicillium* (65%), and *Trichoderma* (36%) (Table 1).

## 4. Discussion

### 4.1. The Advantages of Direct Fungal Colony PCR

We found the direct fungal colony PCR technique presented here to be fast and easy to handle, allowing for DNA amplification directly from fungal tissue without prior manipulation or treatment; instead, the mycelium is recovered directly from culture plates or other substrates with a sterile needle or toothpick, and used for direct PCR. This method, thus, requires neither the use of expensive and specialized equipment, nor of special kits or reagents.

Our direct colony PCR technique worked for a wide range of soil hyphomycete taxa, and was also always very successful for yeasts. Compared to commercially available kits, this technique is cheaper, and can be carried out anywhere, also under circumstances where access to commercial kits is difficult or too expensive. In addition, we suggest that this technique may be a valuable tool for teaching courses, where the robustness of techniques used as well as time and money are of immediate concern.

The main advantage of this direct fungal colony PCR method compared to established direct PCR protocols for fungi is that it does not require time-consuming previous tissue manipulation or the use of expensive reagents such as proteinase K or other enzymes. The only additional reagent used for direct fungal colony PCR is bovine serum albumin (BSA). However, pre-treatment of fungal tissue, as earlier described by Pancher *et al.* [16], is still the most promising strategy for fungal colonies belonging to genera that could not be successfully (or at least reliably) amplified by direct fungal colony PCR, e.g., *Trichoderma* or *Absidia* spp. For this pre-treatment, fresh mycelium and the agar medium underneath are frozen at −80 °C and lysed mechanically. Then, sterile distilled water is added to the lysate, which is then mixed and centrifuged. Finally, the supernatant is used as a template [16]. Alternatively, fungal tissue could also be pre-treated with heat, buffers, microwave, and enzymes [12].

The direct colony PCR method discussed here proved very suitable to obtain sequences from a wide range of soil hyphomycete isolates belonging to different phylogenetic lineages (Ascomycota, Basidiomycota, and Zygomycota), among them important and widespread genera of saprobial soil fungi like *Geomyces*/*Pseudogymnoascus*, *Cladosporium*, and *Mortierella*. The very high overall PCR success obtained in this study suggests broad applicability for this fast, cheap, and reliable technique. This direct PCR technique was established based on the excellent results obtained by direct PCR of ectomycorrhizal tissues [14,17] and was also successfully applied on pure cultures of a range of agaricoid and polyporous fungi [20]. This suggests that this PCR method would also work for other fungal groups, which were not included in the test e.g., food-borne fungi or plant-pathogenic fungi. 

### 4.2. Factors Affecting Direct Colony PCR Success

Taxonomic affiliation affects direct colony PCR success: The direct PCR technique can be recommended for a cheap, high-throughput amplification technique for fungal cultures covering a wide taxonomic range, because overall PCR success was very high (86%). However, direct colony PCR success varied between genera of hyphomycetes. Most of the tested genera of soil-borne hyphomycetes like *Cladosporium*, *Geomyces*, *Fusarium*, and *Mortierella* could be amplified with high success, and soil-borne yeasts were always successfully amplified. Other fungal growth forms like coelomycetous or as sterile mycelia also appear to be very suitable for direct colony PCR. *Mucor*, *Trichoderma*, and *Penicillium* had noticeably lower PCR success in comparison with other fungal groups that were repeatedly tested, and DNA could not be amplified from *Absidia* isolates (seven different isolates, all repeatedly tested). A pre-treatment of fungal tissue or spores, e.g., as described by Pancher *et al.* [16] seems to be necessary for successful direct colony PCR of these fungal genera.

Failed PCR reactions could also be caused by excessive amounts of fungal template material added to the PCR master mix [14]. Transferring only miniscule amounts of fungal tissue into the reaction mixture is critical for success, but can prove challenging when working with isolates that show excessive sporulation (e.g., *Penicillium*) and/or extremely fast growth (*Mucor* and *Absidia*).

DNA template quality is usually good for fungal samples obtained from the growing edge of fungal colonies: DNA is neither fragmented nor degraded. However, DNA purity can be an important issue for PCR success, as shown for plants [27]. Polysaccharides and pigments impair DNA purity, and have been described as an important issue in PCR amplification of *Trichoderma* [28]. In these cases, DNA extraction and DNA purification are therefore essential steps for a successful PCR amplification.

Finally, primer choice can sometimes be crucial for PCR success [29], and potential primer bias is an issue also for fungi [30]. Multiple direct colony PCRs with different primer combinations or specific primers [31,32,33,34,35] could be carried out to solve this problem.

### 4.3. Potential Applications for Direct Fungal Colony PCR

This fast and cheap direct fungal colony PCR method can be used for many other applications apart from obtaining barcoding sequences from pure culture collections. Direct colony PCR products can also be used for cloning and thus allow e.g. for a direct amplification of fungi from the environment without prior cultivation. The use of other primers and primer combinations enables for a fast and easy amplification of other target genes. Direct fungal colony PCR also allows for a reliable screening of fungal isolates, e.g. for mutant strains. A faster and cheaper method for PCR amplification of fungal environmental isolates will also contribute to a better knowledge concerning the ecology and biogeography of fungi, and to the discovery of potentially novel fungal taxa.

## 5. Conclusions

Direct fungal colony PCR is a fast and reliable method for crude mycelium-based amplification of the ITS1-5.8S-ITS2 region of the fungal ribosomal DNA cluster. PCR success rate is generally high. A broad application of this method should lead to a simplification of molecular taxonomic analyses, and will allow for more extensive, sequence-based analyses of fungal environmental isolates. Improved techniques for an accelerated DNA barcode reference library construction will result in considerably improved sequence databases covering a wider taxonomic range. Fast, cheap, and reliable methods for obtaining DNA sequences from fungal isolates are, therefore, a valuable tool for the scientific community.

## Figures and Tables

**Table 1:** Relative direct PCR success for genera of soil fungi (in alphabetical order) with taxonomic affiliations, MOTUs obtained within the genus and number of fungal isolates tested.

Genus/Name	Taxonomic Affiliation	MOTUs	Tested Isolates	PCR-Success (%)
*Absidia*	*Mucoromycotina*	0	7	0
*Aureobasidium*	*Dothideomycetes*	1	2	100
*Bjerkandera*	*Agaricomycotina*	1	1	100
*Botrytis/Sclerotinia*	*Leotiomycetes*	1	2	100
*Cadophora*	*Leotiomycetes*	1	1	100
*Chaetosphaeronema*	*Dothideomycetes*	1	1	100
*Cladosporium/Davidiella*	*Dothideomycetes*	5	63	89
*Cryptococcus*	Mitosporic Basidiomycetes	10	67	100
*Cystodendron*	*Leotiomycetes*	1	1	100
*Cystofilobasidium*	*Agaricomycotina*	1	1	100
*Didymella*	*Dothideomycetes*	1	6	100
*Dioszegia*	*Agaricomycotina*	2	3	100
*Drechslera*	*Dothideomycetes*	1	1	100
*Epicoccum*	*Dothideomycetes*	1	1	100
*Fusarium/Gibberella*	*Sordariomycetes*	1	33	85
*Geomyces/Pseudogymnoascus*	*Leotiomycetes*	8	147	99
*Guehomyces*	*Agaricomycotina*	1	2	100
*Helgardia*	*Leotiomycetes*	1	1	100
Helotiales unknown	*Leotiomycetes*	2	3	100
*Herpotrichia*	*Dothideomycetes*	4	5	100
*Holtermaniella*	*Agaricomycotina*	1	1	100
*Ilyonectria*	*Sordariomycetes*	1	1	100
*Leptodontidium*	*Leotiomycetes*	1	1	100
*Leuconeurospora*	*Leotiomycetes*	1	2	100
*Leucosporidiella/-ium*	*Pucciniomycotina*	2	5	100
*Monodictys*	*Sordariomycetes*	1	1	100
*Monographella/Microdochium*	*Sordariomycetes*	3	17	94
*Mortierella*	*Mortierellomycotina*	13	112	89
*Mrakia*	*Agaricomycotina*	2	3	100
*Mrakiella*	*Agaricomycotina*	1	2	100
*Mucor*	*Mucoromycotina*	6	48	58
*Neonectria*	*Sordariomycetes*	1	1	100
*Paraconiothyrium*	*Dothideomycetes*	1	1	100
*Penicillium*	*Eurotiomycetes*	9	105	65
*Phacidium*	*Leotiomycetes*	2	45	96
*Phaeosphaeria*	*Dothideomycetes*	1	2	100
*Phoma*	*Dothideomycetes*	2	9	100
*Rhodotorula*	Mitosporic Basidiomycetes	1	6	100
*Seimatosporium*	*Sordariomycetes*	1	2	100
*Stagonosporopsis*	*Dothideomycetes*	1	2	100
*Sydowia*	*Dothideomycetes*	1	2	100
*Tetracladium*	*Leotiomycetes*	3	3	100
*Thelebolus*	*Leotiomycetes*	1	1	100
*Trichoderma/Hypocrea*	*Sordariomycetes*	2	22	36
*Trichosporon*	Mitosporic Basidiomycetes	1	2	100
*Truncatella*	*Sordariomycetes*	2	9	100
*Umbelopsis*	*Mucoromycotina*	7	32	94
Unknown sterile mycelia	Unknown	3	5	60

## Abbreviations

The following abbreviations are used in this manuscript:

BSA	bovine serum albumin
CYA	Czapek yeast extract agar
DNA	deoxyribonucleic acid
G25N	25% glycerol nitrate agar
ITS	internal transcribed spacer
JMRC	Jena Microbial Resource Collection
MEA	malt extract agar
MOTU	molecular operational taxonomic unit
PCR	polymerase chain reaction

## Appendix

**Table A1:** List of MOTUs obtained with direct colony PCR from 788 fungal strains. GenBank accession numbers (ACCN) and collection numbers in the Jena Microbial Resource Collection (JMRC:SF:Nr) are provided. MOTUs are sorted alphabetically by description.

MOTU ID	MOTU Description	GenBank ACCN	JMRC:SF:Nr
MK_42	*Aureobasidium* sp.	KP714635	JMRC:SF:12047
GW_52	*Bjerkandera adusta*	KP714580	JMRC:SF:12006
GW_54	*Botrytis* sp.	KP714582	JMRC:SF:12008
GW_07	*Cladosporium* sp. 1	KP714536	JMRC:SF:11967
GW_17	*Cladosporium* sp. 2	KP714546	JMRC:SF:11977
GW_43	*Cladosporium* sp. 3	KP714571	JMRC:SF:12000
GW_59	*Cladosporium* sp. 4	KP714587	JMRC:SF:12012
MK_40	*Cladosporium* sp. 5	KP714642	JMRC:SF:12052
GW_40	*Cryptococcus* aff. *albidosimilis*	KP714568	JMRC:SF:11998
GW_58	*Cryptococcus* aff. *victoriae*	KP714586	-
MK_35	*Cryptococcus* *friedmannii*	KP714628	JMRC:SF:12041
GW_18	*Cryptococcus* sp. 1	KP714547	JMRC:SF:11978
GW_24	*Cryptococcus* sp. 2	KP714553	JMRC:SF:11984
GW_33	*Cryptococcus* sp. 3	KP714562	JMRC:SF:11992
GW_36	*Cryptococcus* sp. 4	KP714565	JMRC:SF:11995
MK_45	*Cryptococcus* sp. 5	KP714638	JMRC:SF:12048
MK_72	*Cryptococcus* sp. 6	KP714662	JMRC:SF:12074
GW_19	*Cryptococcus terricola*	KP714548	JMRC:SF:11979
MK_53	*Cryptococcus victoriae*	KP714646	JMRC:SF:12056
MK_75	*Cystofilobasidium infirmominiatum*	KP714665	JMRC:SF:12077
MK_14	*Davidiella* sp. 1	KP714607	JMRC:SF:12027
MK_70	*Davidiella* sp. 2	KP714660	JMRC:SF:12072
MK_10	*Dioszegia* sp. 1	KP714603	JMRC:SF:12024
MK_57	*Dioszegia* sp. 2	KP714649	JMRC:SF:12060
GW_48	Dothideomycetes unknown	KP714576	JMRC:SF:12003
GW_35	*Drechslera* sp.	KP714564	JMRC:SF:11994
GW_63	*Epicoccum* sp.	KP714591	JMRC:SF:12016
GW_09	*Fusarium* sp. 1	KP714538	JMRC:SF:11969
MK_24	*Fusarium* sp. 2	KP714617	JMRC:SF:12032
MK_06	*Geomyces* aff. *vinaceus*	KP714599	JMRC:SF:12022
MK_05	*Geomyces* *pannorum* 1	KP714598	JMRC:SF:12021
MK_20	*Geomyces* *pannorum* 2	KP714613	JMRC:SF:12030
GW_02	*Geomyces* sp. 1	KP714531	JMRC:SF:11962
GW_03	*Geomyces* sp. 2	KP714532	JMRC:SF:11963
GW_53	*Geomyces* sp. *3*	KP714581	JMRC:SF:12007
MK_61	*Geomyces* sp. *4*	KP714653	JMRC:SF:12064
MK_38	*Guehomyces pullulans*	KP714631	JMRC:SF:12043
GW_46	*Helgardia* sp.	KP714574	JMRC:SF:12001
MK_09	*Helotiales unknown* 1	KP714602	JMRC:SF:12023
MK_39	*Helotiales unknown* 2	KP714632	JMRC:SF:12044
GW_42	*Herpotrichia juniperi* 1	KP714570	-
MK_32	*Herpotrichia juniperi* 2	KP714625	JMRC:SF:12038
MK_46	*Herpotrichia juniperi* 3	KP714639	JMRC:SF:12049
GW_39	*Hormonema* sp.	KP714567	JMRC:SF:11997
GW_65	*Ilyonectria* sp.	KP714593	JMRC:SF:12018
MK_63	*Leptodontidium orchidicola*	KP714654	JMRC:SF:12066
MK_01	*Leucosporidiella* sp.	KP714594	JMRC:SF:12019
GW_34	*Leucosporidium* sp.	KP714563	JMRC:SF:11993
GW_12	*Monographella aff. lycopodina*	KP714541	JMRC:SF:11972
GW_50	*Monographella* sp.	KP714578	JMRC:SF:12005
GW_13	*Mortierella* aff. *gamsii*	KP714542	JMRC:SF:11973
MK_29	*Mortierella alpina* 1	KP714622	JMRC:SF:12035
MK_34	*Mortierella alpina* 2	KP714627	JMRC:SF:12040
MK_77	*Mortierella alpina* 3	KP714667	JMRC:SF:12079
MK_52	*Mortierella antarctica*	KP714645	JMRC:SF:12055
MK_50	*Mortierella globulifera* 1	KP714643	JMRC:SF:12053
MK_54	*Mortierella globulifera* 2	KP714647	JMRC:SF:12057
GW_08	*Mortierella humilis*	KP714537	JMRC:SF:11968
GW_29	*Mortierella macrocystis*	KP714558	JMRC:SF:11988
GW_01	*Mortierella* sp. 1	KP714530	JMRC:SF:11961
GW_16	*Mortierella* sp. 2	KP714545	JMRC:SF:11976
GW_20	*Mortierella* sp. 3	KP714549	JMRC:SF:11980
GW_27	*Mortierella* sp. 4	KP714556	-
MK_31	*Mrakia blollopsis*	KP714624	JMRC:SF:12037
MK_41	*Mrakia* sp.	KP714634	JMRC:SF:12046
MK_25	*Mrakiella aquatica*	KP714618	JMRC:SF:12033
GW_44	*Mucor* aff. *abundans*	KP714572	-
GW_45	*Mucor flavus*	KP714573	-
GW_15	*Mucor hiemalis* 1	KP714544	JMRC:SF:11975
MK_15	*Mucor hiemalis* 2	KP714608	JMRC:SF:12028
MK_69	*Mucor hiemalis* 3	KP714659	JMRC:SF:12071
GW_47	*Mucor strictus*	KP714575	JMRC:SF:12002
MK_27	Nectriaceae unknown	KP714620	JMRC:SF:12034
GW_55	*Penicillium* aff. *brevicompactum*	KP714583	JMRC:SF:12009
GW_14	*Penicillium* aff. *lividum*	KP714543	JMRC:SF:11974
GW_31	*Penicillium* aff. *melinii*	KP714560	JMRC:SF:11990
GW_10	*Penicillium* aff. *spinulosum*	KP714539	JMRC:SF:11970
GW_25	*Penicillium* aff. *ubiquetum*	KP714554	JMRC:SF:11985
GW_04	*Penicillium* sp. 1	KP714533	JMRC:SF:11964
GW_23	*Penicillium* sp. 2	KP714552	JMRC:SF:11983
GW_32	*Penicillium* sp. 3	KP714561	JMRC:SF:11991
GW_49	*Penicillium* sp. 4	KP714577	JMRC:SF:12004
GW_64	*Penicillium* sp. 5	KP714592	JMRC:SF:12017
MK_60	*Penicillium* sp. 6	KP714652	JMRC:SF:12063
GW_06	*Phacidium* aff*. pseudophacidioides*	KP714535	JMRC:SF:11966
GW_05	*Phacidium* aff. *trichophori*	KP714534	JMRC:SF:11965
GW_41	*Phaeosphaeria* sp.	KP714569	JMRC:SF:11999
GW_56	*Pleosporales unknown* 1	KP714584	JMRC:SF:12010
MK_13	*Pleosporales unknown* 2	KP714606	JMRC:SF:12026
MK_36	*Pleosporales unknown* 3	KP714629	JMRC:SF:12042
MK_47	*Pleosporales unknown* 4	KP714640	JMRC:SF:12050
MK_30	*Pseudeurotiaceae* sp.	KP714623	JMRC:SF:12036
MK_40	*Pseudogymnoascus destructans* 1	KP714633	JMRC:SF:12045
MK_51	*Pseudogymnoascus destructans* 2	KP714644	JMRC:SF:12054
MK_56	*Pseudogymnoascus destructans* 3	KP714648	JMRC:SF:12059
MK_33	*Rhodotorula colostri*	KP714626	JMRC:SF:12039
GW_26	*Rhodotorula* sp.	KP714555	JMRC:SF:11986
GW_51	*Stemphylium* sp.	KP714579	-
GW_57	*Stereum sanguinolentum*	KP714585	JMRC:SF:12011
MK_21	*Sterile Mycelium (Ascomycete)* 1	KP714614	JMRC:SF:12031
MK_59	*Sterile Mycelium (Ascomycete)* 2	KP714651	JMRC:SF:12062
MK_65	*Sterile Mycelium (Ascomycete)* 3	KP714655	JMRC:SF:12067
MK_66	*Sterile Mycelium (Ascomycete)* 4	KP714656	JMRC:SF:12068
MK_73	*Sterile Mycelium (Ascomycete)* 5	KP714663	JMRC:SF:12075
MK_74	*Sterile Mycelium (Ascomycete)* 6	KP714664	JMRC:SF:12076
MK_76	*Sterile Mycelium (Ascomycete)* 7	KP714666	JMRC:SF:12078
MK_48	*Sterile Mycelium (Basidiomycete)* 1	KP714641	JMRC:SF:12051
MK_67	*Sterile Mycelium (Basidiomycete)* 2	KP714657	JMRC:SF:12069
MK_68	*Sterile Mycelium (Basidiomycete)* 3	KP714658	JMRC:SF:12070
MK_03	*Tetracladium* sp. 1	KP714596	JMRC:SF:12020
MK_17	*Tetracladium* sp. 2	KP714610	JMRC:SF:12029
MK_71	*Tetracladium* sp. 3	KP714661	JMRC:SF:12073
MK_58	*Thelebolus* sp.	KP714650	JMRC:SF:12061
GW_22	*Trichoderma* sp. 1	KP714551	JMRC:SF:11982
GW_62	*Trichoderma* sp. 2	KP714590	JMRC:SF:11996
GW_38	*Trichoderma* sp. 3	KP714566	JMRC:SF:12015
MK_12	*Truncatella angustata*	KP714605	JMRC:SF:12025
GW_11	*Umbelopsis* sp. 1	KP714540	JMRC:SF:11971
GW_21	*Umbelopsis* sp. 2	KP714550	JMRC:SF:11981
GW_28	*Umbelopsis* sp. 3	KP714557	JMRC:SF:11987
GW_30	*Umbelopsis* sp. 4	KP714559	JMRC:SF:11989
GW_60	*Umbelopsis* sp. 5	KP714588	JMRC:SF:12013
GW_61	*Umbelopsis* sp. 6	KP714589	JMRC:SF:12014
